# Reversing Epithelial Polarity in Pluripotent Stem Cell-Derived Intestinal Organoids

**DOI:** 10.3389/fbioe.2022.879024

**Published:** 2022-04-25

**Authors:** Panagiota Kakni, Carmen López-Iglesias, Roman Truckenmüller, Pamela Habibović, Stefan Giselbrecht

**Affiliations:** ^1^ Department of Instructive Biomaterials Engineering, MERLN Institute for Technology-Inspired Regenerative Medicine, Maastricht University, Maastricht, Netherlands; ^2^ Microscopy CORE Lab, Maastricht Multimodal Molecular Imaging Institute (M4I), Maastricht University, Maastricht, Netherlands

**Keywords:** epithelial organoids, intestinal organoids, apicobasal polarity, reversed polarity, advanced 3D models

## Abstract

The inner surface of the intestine is a dynamic system, composed of a single layer of polarized epithelial cells. The development of intestinal organoids was a major breakthrough since they robustly recapitulate intestinal architecture, regional specification and cell composition *in vitro*. However, the cyst-like organization hinders direct access to the apical side of the epithelium, thus limiting their use in functional assays. For the first time, we show an intestinal organoid model from pluripotent stem cells with reversed polarity where the apical side faces the surrounding culture media and the basal side faces the lumen. These inside-out organoids preserve a distinct apico-basolateral orientation for a long period and differentiate into the major intestinal cell types. This novel model lays the foundation for developing new *in vitro* functional assays particularly targeting the apical surface of the epithelium and thus offers a new research tool to study nutrient/drug uptake, metabolism and host-microbiome/pathogen interactions.

## Introduction

The intestinal epithelium is a highly organized, self-renewing tissue mainly serving two roles. First, it forms a physical barrier to avoid the crossing of harmful substances in the intestinal lumen and second, it regulates the nutrient absorption and metabolism. Within this simple columnar epithelial layer, the establishment and maintenance of cell polarity with distinct apical and basolateral surfaces is considered crucial for the proper tissue development and function. Each of these compartments has a different structure, function and macromolecule composition (Klunder et al., 2017). The apical surface faces the lumen and is responsible for the absorption of nutrients while the basolateral surface faces the stroma and mediates nutrient transport. Apart from the apico-basolateral polar organization, the differentiation towards the major intestinal cell types (enterocytes, Paneth cells, goblet cells etc.) is of utmost importance for the proper functioning of the intestine. Various cell lines and animal models have been utilized to model the human intestinal epithelium but the full complexity of it has not yet been accurately recapitulated *in vitro*.

Advances in stem cell research made it possible to create *in vitro* 3D organ-like structures from either adult or pluripotent stem cells that better recapitulate the *in vivo* tissues than traditional 2D cell culture models. The generation of intestinal organoids was a major research breakthrough, yielding a new tool to study the intestinal epithelium (Sato et al., 2009; Sato and Clevers, 2013; Clevers, 2016). The culture of intestinal organoids is a relatively simple process, requiring a tailored cell culture medium and hydrogels (e.g., basement membrane matrix secreted by Engelbreth-Holm-Swarm mouse sarcoma cells) serving as an extracellular matrix (ECM) substitute (Fatehullah et al., 2016). The resulting 3D multicellular constructs demonstrate an *in vivo*-like architecture with crypt-villus structures surrounding a central lumen and contain both proliferating and differentiated cell types. However, the enclosed position of the lumen hinders access to the apical surface of the epithelium, thus limiting studies related to nutrient uptake and host-microbiome/pathogen interactions. To overcome this, three different approaches have been taken so far. The first is the use of microinjection techniques where microbes or other infectious agents are injected directly to the lumen of organoids (Bartfeld et al., 2015; Hill et al., 2017; Williamson et al., 2018). This is a labor-intensive, time-consuming and often even disruptive process. The second is the formation of 2D cell monolayers by dissociating organoids (VanDussen et al., 2015; Kozuka et al., 2017; Wang et al., 2017; Altay et al., 2019). Although, in this way, access to both the apical and basolateral sides is granted, the 3D tissue-like structure of the organoids is lost thus making the system less physiologically relevant. Finally, the third method is the establishment of organoid models with reversed polarity (Co et al., 2019; Co et al., 2021; Nash et al., 2021; Stroulios et al., 2021). In this case, the apical surface of the epithelium is facing the cell culture media thus allowing direct access to it. This method has been applied to human (Co et al., 2019; Co et al., 2021; Stroulios et al., 2021), porcine (Li et al., 2020) and chicken (Nash et al., 2021) primary cell-derived intestinal organoids.

Here, we report the development of an intestinal organoid model with reversed polarity using pluripotent stem cells (PSCs). Following a stepwise directed differentiation protocol, we generated organoids consisting of a simple columnar epithelium patterned into crypt-like and villus-like structures. They contain the major intestinal differentiated cell types and are surrounded by a mesenchymal compartment. In our novel microwell-based culture protocol, the original embedding of organoids in a solid matrix was replaced by a suspension system, which allowed for a uniform, long-term reversal of the epithelial polarity. These novel pluripotent stem cell-derived apical-out organoids are a powerful new tool for studies relating but not limited to infectious diseases, gut microbiota, nutrient absorption and drug metabolism.

## Materials and Methods

### Maintenance of PSCs

The human embryonic stem cell line WA09 (H9) was obtained from WiCell and the induced pluripotent stem cell line iPSC72_3 was obtained by the Pluripotent Stem Cell Facility at Cincinnati Children’s Hospital Medical Center. ES and iPS cell lines were maintained in feeder-free conditions on Matrigel (Corning®) using mTESR®1 (StemCell Technologies). Colonies were passaged every four to 5 days depending on colony density using Gentle Cell Dissociation reagent (StemCell Technologies).

### Fabrication and Preparation of Microwell Arrays

Polymer film based microwell arrays were fabricated by microthermorming as described previously (Giselbrecht et al., 2006; Kakni et al., 2020). Every array accommodated 289 U-bottomed microwells and each microwell had a diameter of 500 μm and a depth of approximately 300 μm. Prior to cell culture, microwell arrays were sterilized in a graded series of 2-propanol (VWR) (100%–70%–50%–25%–10%) and then washed twice with Dulbecco’s phosphate buffered saline (PBS; Sigma-Aldrich). Subsequently, they were placed at the bottom of non-treated 24-well plates, where they were kept in place by elastomeric O-rings (ERIKS).

### Differentiation of PSCs to Definitive Endoderm and Hindgut in Microwells

The protocol for directed differentiation of intestinal organoids was carried out as previously described (Spence et al., 2011) with small modifications. PSCs were dissociated into single cells using TrypLE™ Express Enzyme (Thermofisher) and seeded on microwell arrays at a density of 1,000 cells/microwell in mTesR1 supplemented with Y-27632 (10 μΜ; Tocris) to create embryoid bodies (EBs). The following 3 days, EBs were treated with Activin A (100 ng/ml; Cell guidance systems) in RPMI 1640 (Thermofisher) medium supplemented with increasing concentrations (0%, 0.2%, 2%) of Hyclone defined fetal bovine serum (dFBS; Fisher scientific). For hindgut specification, the DE spheroids were treated with a combination of FGF4 (500 ng/ml; R&D Systems) and CHIR99021 (3 μM; Stemgent) for four additional days. The medium was exchanged daily. In order to avoid the removal of the spheroids from the microwells, the plate was slightly tilted and the medium was aspirated from the sidewalls.

### Differentiation Towards Intestinal Organoids

For the apical-in intestinal organoids, hindgut spheroids were collected, suspended in 50 μl Matrigel and plated as droplets into tissue culture treated 24-well plates. After letting the Matrigel solidify at 37°C and 5% CO_2_ for 15 min, the Matrigel drops containing the spheroids, were overlaid with Advanced DMEM/F-12 supplemented with B27, N2, Hepes, penicillin/streptomycin, L-glutamine (all Thermofisher), EGF (50 ng/ml; R&D systems), Noggin (100 ng/ml; R&D systems) and R-Spondin (500 ng/ml; R&D systems). The medium was refreshed every 4 days.

For the apical-out intestinal organoids, on day 8 the hindgut spheroids were placed in suspension culture in non-tissue culture-treated 6-well plates (plates with different sizes can be used as well). To avoid surface-cell adherence, the plates were coated with 1% Pluronic solution in PBS (Sigma-Aldrich) for 2 h at 37°C and then washed two times with PBS. The medium used, had the same composition as the apical-in organoids, but in this case Matrigel was added as a medium supplement at a concentration of 2%.

### Immunofluorescence and Confocal Microscopy

EBs, DE spheroids, hindgut spheroids and intestinal organoids were fixed with 4% paraformaldehyde (VWR) in PBS for 30 min. Following that, permeabilization was performed with 0.5% Triton X-100 (Merck) in PBS for another 30 min at room temperature (RT). Blocking was performed with 5% donkey serum (VWR) in permeabilization solution for 30 min at RT as well. Afterwards, primary antibodies were incubated overnight at 4°C and the next day secondary antibodies were added for 2 h at RT. Finally, samples were counterstained with 4′,6-diamidino-2-phenylindole (DAPI) (Sigma-Aldrich) and mounted with Lab Vision PermaFluor Aqueous Mounting Medium (Thermofisher). A full list of antibodies is provided in the Supplementary Material. For the imaging of the immunostained samples, a confocal laser scanning microscopy (Leica TCS SP8) was utilized and the images were processed with ImageJ. Quantification was performed using the open access software QuPath.

### RNA Isolation and Quantitative Real-Time PCR (qPCR)

Organoids were collected and the total RNA was extracted using the RNeasy Mini Kit (Qiagen) according to the manufacturer instructions. For the cDNA synthesis, the iScript cDNA Synthesis Kit (Bio-Rad) was utilized. Finally, qPCR was carried out using the iQ SYBR Green Supermix (Bio-Rad), on a CFX96 Real-Time PCR Detection System (Bio-Rad). Gene expression for each sample was normalized using the glyceraldehyde-3-phosphatedehydrogenase (*GAPDH*) or the hypoxanthine phosphoribosyltransferase (*HPRT*) housekeeping genes. *GAPDH* was used for DE and hindgut spheroids, whereas *HPRT* for intestinal organoids. The expression of *GAPDH* could be affected by the different oxygen levels (Caradec et al., 2010) we expect between hydrogel embedded and suspension organoids, thus we chose to use *HPRT* for these samples. Data analysis followed the 2^−ΔΔCt^ method. The results are representative of three independent experiments. The primer sequences are listed in the Supplementary Material.

### Scanning Electron Microscopy (SEM)

Organoids were chemically fixed for 3 h at room temperature with 1.5% glutaraldehyde in 0.067 M cacodylate buffered to pH 7.4 and 1% sucrose. Then they were washed with 0.1 M cacodylate buffer and postfixed with 1% osmium tetroxide in the same buffer containing 1.5% potassium ferricyanide for 1 h in the dark at 4°C. After rinsing with MQ, organoids were dehydrated at RT in a graded ethanol series (70, 90, up to 100%). Then, organoids were dried using HMDS (Hexamethyldisilazane) (>99.9%, Sigma Aldrich, Germany). After HMDS treatment, the samples were mounted on SEM stubs, coated with a thin layer of gold by a sputter coater SC7620 (Quorum Technologies, United Kingdom) and examined with the electron microscope (Jeol JSM-IT200, Japan).

### Transmission Electron Microscopy (TEM)

Organoids were chemically fixed for 3 h at room temperature with 1.5% glutaraldehyde in 0.067 M cacodylate buffered to pH 7.4 and 1% sucrose. Then they were washed with 0.1 M cacodylate buffer and postfixed with 1% osmium tetroxide in the same buffer containing 1.5% potassium ferricyanide for 1 h in the dark at 4°C. After rinsing with MQ, organoids were dehydrated at RT in a graded ethanol series (70, 90, up to 100%), infiltrated with Epon, embedded in the same resin and polymerized for 48 h at 60°C. Ultrathin sections of 60 nm were cut using a diamond knife (Diatome) on a Leica UC7 ultramicrotome, and transferred onto 50 Mesh copper grids covered with a Formvar and carbon film. Sections were stained with 2% uranyl acetate in 50% ethanol and lead citrate. Then, sections were observed in a Tecnai T12 Electron Microscope equipped with an Eagle 4kx4k CCD camera (Thermo Fisher Scientific, Netherlands) or Veleta 2kx2k CCD camera (Olympus Soft Imaging, Germany).

### Statistical Analysis

All statistical analysis was performed using GraphPad Prism 9 software. Student’s two-tailed *t*-test with Welch’s correction (two groups) or one-way ANOVA followed by Tukey’s test (> two groups) were used to determine statistical significance. Significant differences were defined as *p* < 0.05. *p* values of statistical significance are represented as ****p < 0.0001, ***p < 0.001, **p < 0.01, and *p < 0.05. Error bars in figures indicate standard error of the mean (S.E.M.).

## Results

### Embryoid Body-Based Differentiation Towards Intestinal Tissue

**FIGURE 1:**
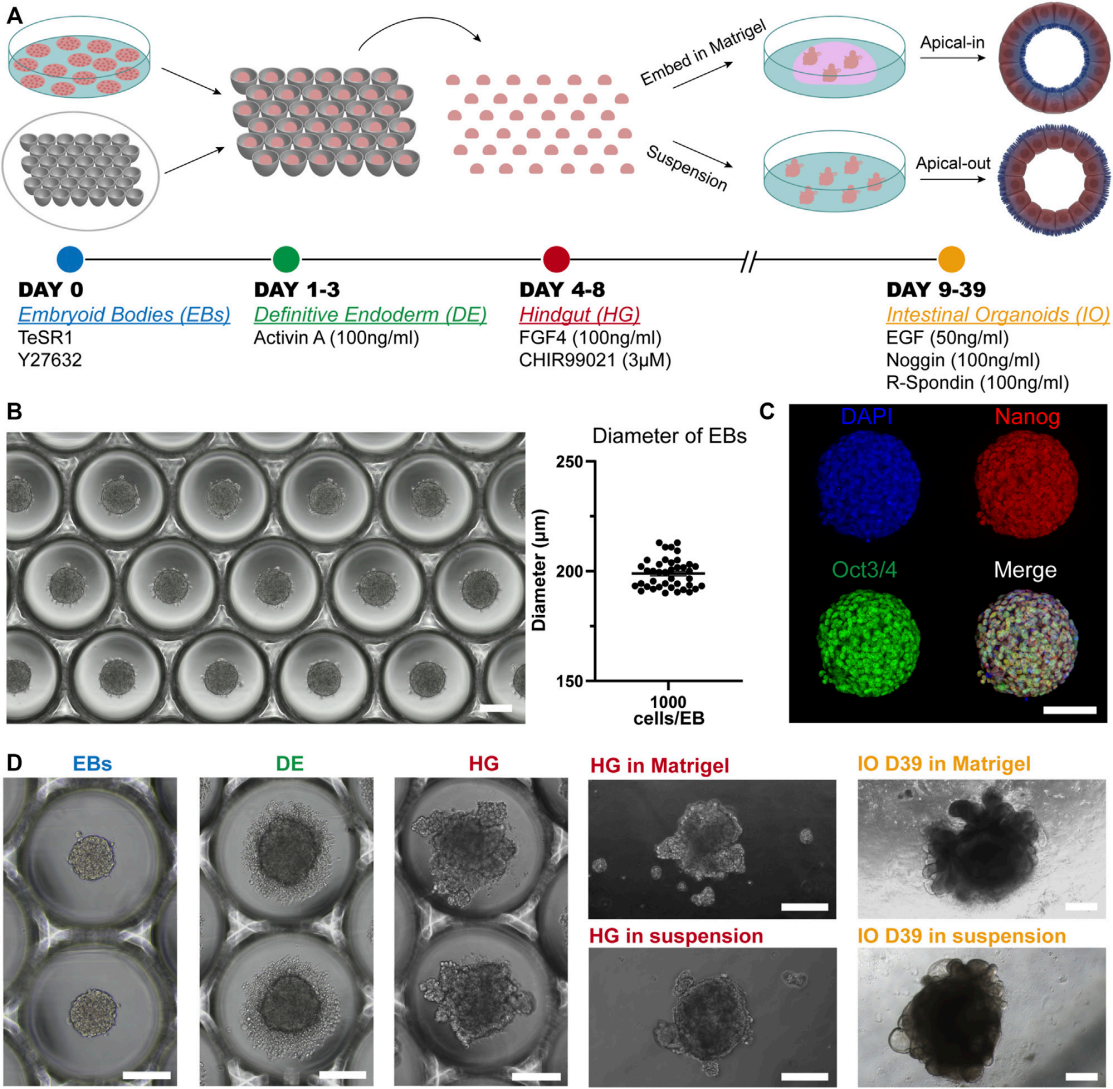
Overview of the *in vitro* culture system established for the directed differentiation of EBs towards intestinal organoids. **(A)** Schematic representation of the protocol. **(B)** Formation of EBs in thermoformed microwell arrays. The graph represents the diameter of EBs 24 h after the cell seeding. Each point displays an individual EB; horizontal line and error bar indicate mean ± S.E.M. (*n* = 3). Scale bar: 200 μm. **(C)** Fluorescence microscopy images of undifferentiated EBs stained for the pluripotency markers Oct3/4 (green) and Nanog (red) and counterstained with DAPI (blue). Scale bar: 100 μm. **(D)** Bright-field images representative of each stage of the differentiation. Scale bars: 200 μm.

The generation of our PSC-derived intestinal organoids is based on the directed differentiation method developed by Spence et al. (2011). Here, the human embryonic and induced pluripotent stem cells were dissociated into single cells and seeded onto microwell arrays in order to promote the formation of uniform embryoid bodies (EBs). Next, these EBs were differentiated stepwise towards intestinal organoids (definitive endoderm→ hindgut→ intestinal organoids) (Figure 1A). The microwell arrays were produced in-house with a custom-made design that fits the needs of our experiments. We identified that around 1,000 cells per EB were adequate for the successful formation of intestinal tissue. These cell aggregates had a diameter of approximately 200 μm (Figure 1B). Aggregates of smaller diameter failed to generate intestinal tissue later on. Additionally, to ensure that our cells remain pluripotent after the formation of EBs, we performed immunofluorescence stainings for the widely used pluripotency markers Oct3/4 and Nanog (Figure 1C). The results showed co-localization of these markers, thus confirming their applicability for downstream differentiation. Initiation of the differentiation within a 2D culture system demands tightly regulated seeding densities and equal distribution of cells around the cell culture plates for the successful differentiation of PSCs towards intestinal organoids (McCracken et al., 2011). This process is very limiting and often fails. Our system overcomes this obstacle since the use of microwells offers a simple method to create uniform 3D EBs that can be used as the starting material for the differentiation towards intestinal tissue.

### Differentiation Towards Definitive Endoderm and Hindgut Specification

**FIGURE 2:**
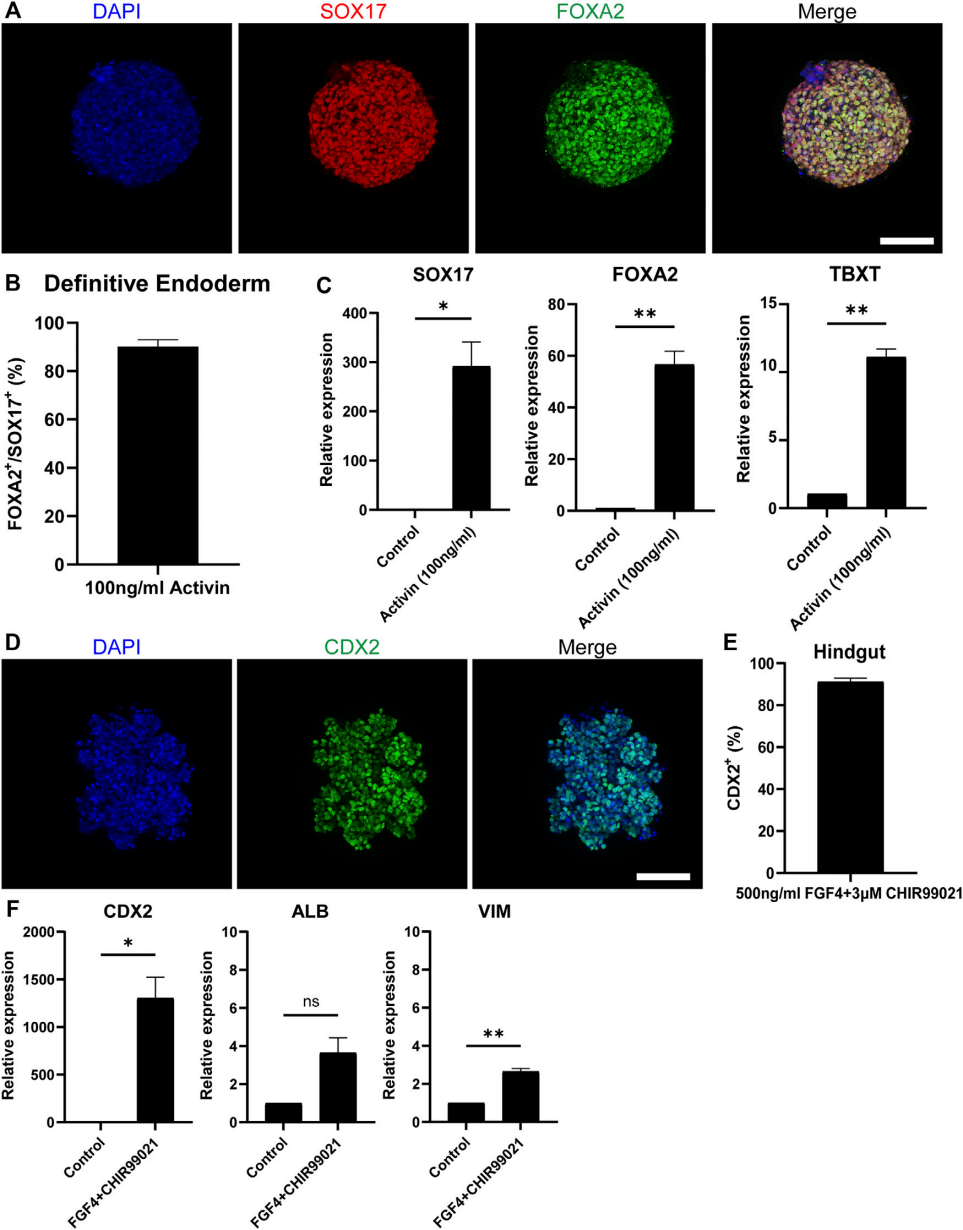
Differentiation towards definitive endoderm followed by hindgut specification. **(A)** H9-derived EBs were treated with 100 ng/ml Activin and the resulting spheroids were stained with the DE markers: SOX17 (red) and FOXA2 (green) and counterstained with DAPI (blue). Scale bar: 100 μm. **(B)** Quantification of the fluorescent images showed that about 92% of the cells in Activin-treated EBs are co-expressing SOX17 and FOXA2. **(C)** qRT-PCR showed significantly increased expression of the DE genes *SOX17* and *FOXA2* and the mesoderm marker *TBXT* but in lower amounts. **(D)** DE spheroids were further treated with FGF4 and CHIR99021 to induce hindgut specification. After 4 days of treatment, the spheroids were stained for the hindgut marker CDX2. Scale bar: 100 μm. **(E)** Quantification of the fluorescent images showed that about 90% of the cells were CDX2^+^. **(F)** qRT-PCR confirmed the robust expression of *CDX2*, whereas there was no significant expression of the foregut marker *ALB*. Low levels of the mesenchymal marker *VIM* were also detected. Error bars indicate mean ± S.E.M. (*n* = 3).

The embryonic development of the intestine initiates during gastrulation when the primary germ layers, the endoderm, the mesoderm and the ectoderm, are formed. Specifically, the intestine derives from the definitive endoderm (DE), which following gastrulation transforms into the primitive gut tube that becomes regionally specified into the foregut, midgut and hindgut along the anterior-posterior axis. After this, the mid- and hindgut will give rise to the intestine (Zorn and Wells, 2009). To generate DE, we treated our EBs in the microwells with Activin-A, which is a nodal-related TGF-β molecule (Figure 1D). After 3 days of treatment, immunofluorescence stainings showed that 90% of the cells in H9-derived DE spheroids (Figures 2A,B) and 92% of the cells in iPSC-derived DE spheroids (Supplementary Figures S1A,B) were co-expressing the known DE markers SRY-Box Transcription Factor 17 (SOX17) and Forkhead Box A2 (FOXA2). Gene expression levels confirmed those results demonstrating a significant increase of *SOX17* and *FOXA2* expression*,* compared to untreated cells, in DE spheroids derived from both cell sources (Figure 2C; Supplementary Figure S1C). We also detected the expression of T-Box Transcription Factor T (*TBXT*), indicating the presence of mesoderm in our cultures, similar to what was previously reported by Spence et al.

To achieve hindgut specification, an appropriate combination of growth factors targeting the Fibroblast Growth Factor (FGF) and Wingless-related integration site (Wnt) pathways is required to repress the foregut and promote the hindgut development (Dessimoz et al., 2006; McCracken and Wells, 2017). For our experiments, we used a combination of FGF4 (500 ng/ml) and CHIR99021 (3 μM). Four days of treatment were adequate to promote the hindgut endoderm specification in our DE spheroids (Figure 1D). The hindgut marker Caudal Type Homeobox 2 (CDX2) was expressed in 91% of the cells in H9-derived hindgut spheroids (Figures 2D,E) and in 90% of the cells in iPSC-derived ones (Supplementary Figures S1D,E). The high levels of *CDX2* expression were confirmed by qPCR (Figure 2F; Supplementary Figure S1F). The foregut marker Albumin (*ALB*) had very low expression without statistical significance, when compared to our undifferentiated controls, while Pancreatic and Duodenal Homeobox 1 (*PDX1*) was not detected. Mesenchyme was identified in our hindgut spheroids using qPCR, as indicated by the expression of Vimentin (*VIM*) (Figure 2F; Supplementary Figure S1F). Overall, our results demonstrate that following a directed differentiation method, EBs can accurately recapitulate both the DE and the hindgut.

### Reversal of Epithelial Polarity in Organoids Cultured in Suspension

**FIGURE 3:**
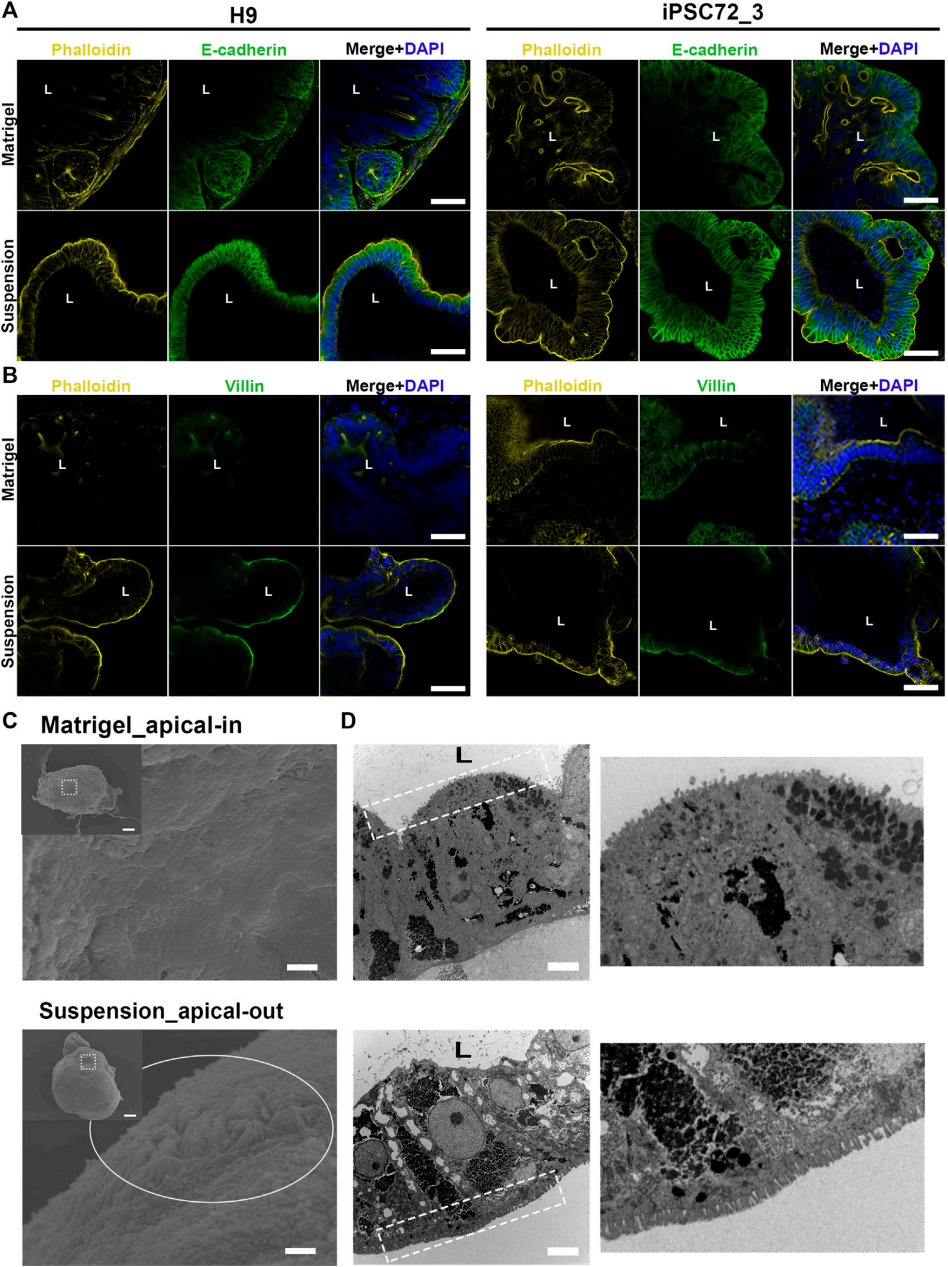
Apico-basolateral organization of human intestinal organoids after 30 days in culture. **(A)** Fluorescent staining of embedded (top) and suspension (bottom) intestinal organoids for the basolateral marker E-cadherin (green) and the apical marker Phalloidin (yellow) shows reversed polarity of the suspension organoids. The left panel demonstrates H9-derived organoids and the right one iPSC72_3-derived organoids. Scale bar: 100 μm. **(B)** These results were confirmed with Villin (green) and Phalloidin (yellow) stainings that were found to co-express in the apical side of the organoids. The left panel demonstrates H9-derived organoids and the right one iPSC-derived organoids. Scale bar: 100 μm. L: lumen. **(C)** SEM was performed in Matrigel embedded (top) and suspension (bottom) organoids showing the presence of microvilli (white circle) in the outer surface of the suspension organoids. Dashed squares represent the area magnified in the corresponding image. Scale bars: 100 μm (inset) and 2 μm. **(D)** TEM indicates that microvilli (white dotted squares) face the lumen in Matrigel embedded (top) organoids, whereas in suspension (bottom) organoids, microvilli face the outer surface. On the right, magnified images demonstrate the microvilli. Scale bars: 5 μm.

The intestinal epithelium is a highly organized tissue and the establishment of proper epithelial polarity is instrumental for balancing the communication between the lumen and surrounding body tissues. In the original protocol for PSC-derived intestinal organoids (Spence et al., 2011), hindgut spheroids are embedded in Matrigel thus leading to the formation of a simple columnar polarized epithelium where the apical surface is facing the enclosed lumen and the basal side the surrounding mesenchyme. In a similar manner, when we embedded our hindgut spheroids in Matrigel (Figure 1D), the resulting organoids demonstrated the same strong apical-basolateral polarity. Specifically, immunofluorescence stainings showed that Phalloidin, which marks the apical side of the organoids, is expressed at the inner side of the organoids facing the lumen, whereas E-cadherin, which marks the basolateral side, is expressed in the outer part facing the culture medium. The immunostainings were performed at days 7, 15, 30 and 50 after embedding the hindgut spheroids in Matrigel for both H9- and iPSC-derived organoids monitoring their structural organization during the whole culture period (Figure 3A; Supplementary Figures S2, S3). In addition, stainings for Villin, a marker of the apical side of the enterocytes, and Phalloidin (Figure 3B) verified our results.

To facilitate the studies of interactions between the epithelium and luminal contents, we aimed to reverse the polarity of our organoids while maintaining their 3D structure. To achieve that, we developed a suspension culture method that allows for hindgut spheroids to mature into intestinal tissue without being embedded in Matrigel (Figures 1A,D). Unlike the original protocol, Matrigel was added at a low concentration to the medium (2%). Already after 7 days, the resulting organoids presented a reversed organization where the apical side was facing the culture medium and the basal side was facing the lumen. Notably, throughout the suspension method, the organoids were grown and matured solely in suspension, whereas in similar protocols the organoids are initially embedded in Matrigel and later on the Matrigel is removed in order to reverse the polarity (Co et al., 2019; Nash et al., 2021). We could show that organoids grown in suspension demonstrate a similar architecture to the Matrigel-embedded ones throughout the culture period, with crypt-villus structures surrounding a central lumen (Supplementary Figure S4). Immunofluorescence stainings evidence the F-actin rich brush border (marked by Phalloidin) and the apical side of the enterocytes (marked by Villin) in the outer part of the organoids thus verifying the polarity reversal at all time-points in both H9- and iPSC-derived organoids (Figures 3A,B; Supplementary Figures S2, S3). Additionally, scanning electron microscopy (SEM) was utilized to verify the two different apico-basolateral organizations. Specifically, the Matrigel-embedded organoids seem to have a “smoother surface,” which is anticipated since the basal side of the organoids is visualized, whereas the suspension organoids exhibit a “rougher surface” in which we can clearly identify the presence of microvilli, further proving that a functional apical side of the organoids is facing outwards (Figure 3C). Finally, the presence of microvilli in reversed positions is illustrated by transmission electron microscopy (TEM; Figure 3D).

To examine the plasticity of these different polarity models, 7 days before reaching full maturation (embedded/suspension day 23), organoids that were initially embedded in Matrigel were placed in suspension culture and organoids that were initially in suspension, were embedded in Matrigel for 1 week. Following this ECM manipulation, the samples were immunofluorescently labelled for E-Cadherin and Phalloidin and the amount of organoids that fully or partially reversed their polarity was quantified (Supplementary Figure S5). Interestingly, none of the organoids (H9- or iPSC-derived) that were isolated from Matrigel and placed in suspension changed their polarity during these 7 days. This indicates that the protocols (Co et al., 2019; Nash et al., 2021) previously described for reversing the polarity of established adult stem cell-derived organoids cannot be applied to reverse the polarity of PSC-derived organoids. We hypothesize that a possibly longer time is required to manipulate the apico-basolateral organization of these organoids. In contrast to this, 90% of H9-derived and 85% of iPSC-derived organoids that were initially in suspension and then embedded in Matrigel reversed their polarity (apical side now facing the lumen). The remaining 10% and 15% of the organoids, respectively, demonstrated an intermediate polarity reversal, where the basal side was facing outwards in some regions of the organoids and in some other regions the apical side was facing outwards, suggesting that the apical-out intestinal organoids are more prone to polarity reversal when embedded in Matrigel.

### Apical-Out Intestinal Organoids Display Various Intestinal Cell Types

**FIGURE 4:**
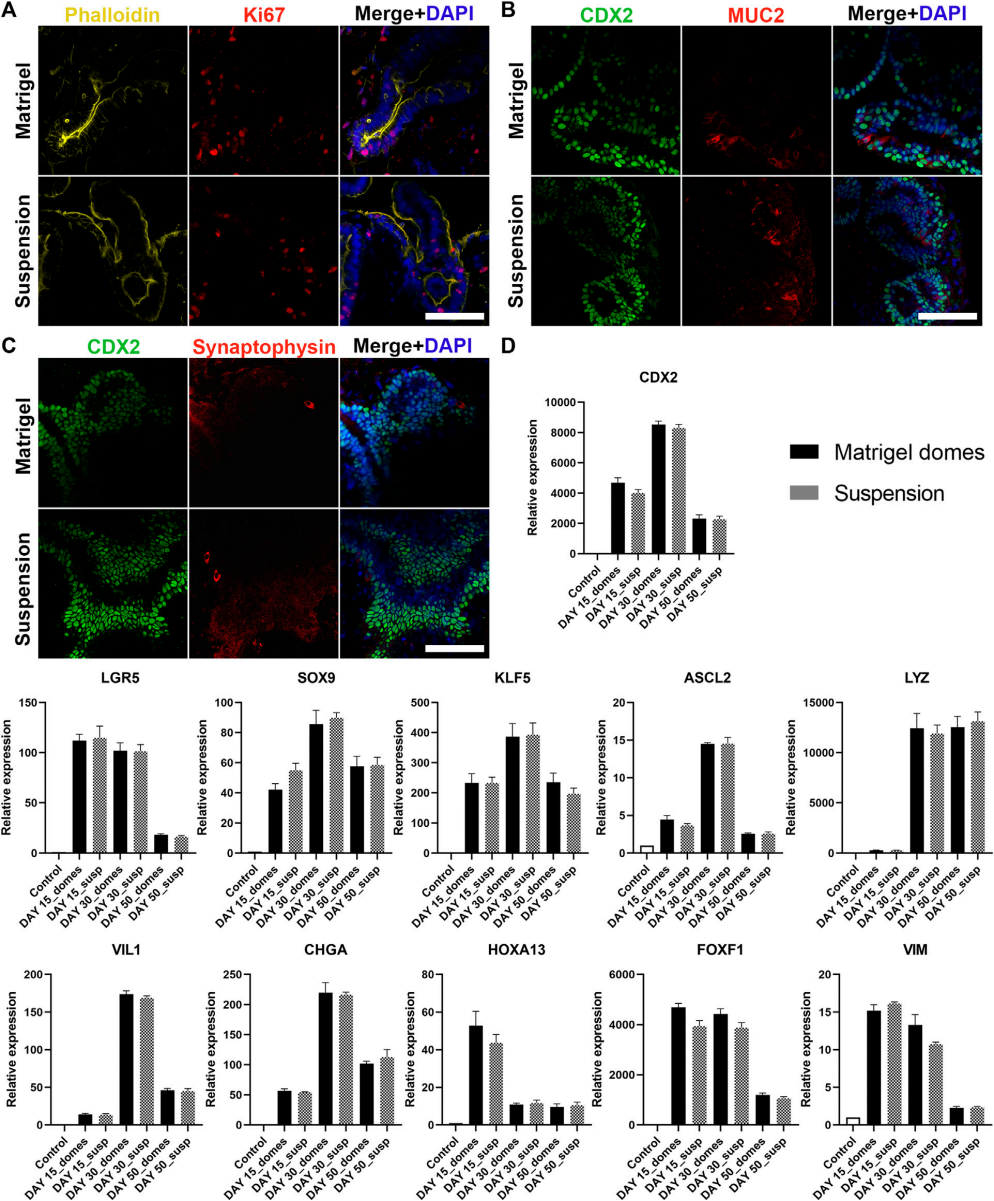
Characterization of H9-derived human intestinal organoids after 30 days in culture. **(A–C)** Immunofluorescence stainings of intestinal markers (Ki67: proliferative cells; CDX2: intestinal transcription factor; MUC2: goblet cells; Synaptophysin: enteroendocrine cells) show similar expression patterns in both embedded and suspension organoids. Scale bars: 100 μm. **(D)** qRT-PCR analysis demonstrates the expression levels of proliferation genes (*LGR5*, *SOX9*, *KLF5*, *ASCL2*), intestinal differentiation genes (*CDX2*, *LYZ*, *VIL1*, *CHGA*, *HOXA13*) and mesenchymal genes (*FOXF1*, *VIM*) after 15, 30 and 50 days in culture. Untreated H9 cells were used as controls. Statistical analysis showed no significant difference between the organoids grown embedded in Matrigel and the organoids grown in suspension at any of the time-points. Error bars indicate mean ± S.E.M. (*n* = 3).

Besides structural organization, the proper function of the intestine is highly dependent on the presence of different intestinal epithelial cell lineages. Indeed, one of the key advantages of organoids as an *in vitro* model is that they can recapitulate to a great extent the cellular diversity of the *in vivo* intestinal epithelium. Therefore, in a next step we verified that our apical-out organoids (derived from H9 or iPSCs) are able to fully mature and differentiate towards the major intestinal cell types. To visualize the intestinal cell differentiation, we used immunofluorescence against the proliferation marker Ki67 and Phalloidin (Figure 4A; Supplementary Figure S6A), the intestinal differentiation marker CDX2, the goblet cell marker Mucin 2 (MUC2) (Figure 4B; Supplementary Figure S6B) and the enteroendocrine marker Synaptophysin (Figure 4C; Supplementary Figure S6C). These stainings were performed at 30 days post Matrigel embedment of hindgut organoids or their transfer to suspension culture. This time-point was selected according to Spence et al. (2011) and Janssen et al. (2020), who demonstrated that intestinal organoids embedded in Matrigel can reach sufficient maturation after approximately 28 days. Organoids from both culture conditions showed similar expression patterns of the mentioned markers. Further characterization of these organoids was performed using relative gene expression quantification. More specifically, the transcriptional expression of multiple intestinal cell lineages was quantified over three developmental stages: days 15, 30 and 50 (Figure 4D; Supplementary Figure S6D). The expression of the intestinal differentiation marker *CDX2*, gradually increased between days 15 and 30, whereas at day 50 a significant decrease was observed. The proliferation markers sex determining region Y-box 9 (*SOX9*), Krueppel-like factor 5 (*KLF5*) and Achaete scute-like 2 (*ASCL2*) showed a similar trend with their peak expression at day 30, whereas leucine-rich repeat-containing G-protein-coupled receptor 5 (*LGR5*) expression peaked at day 15 and was reduced at later time-points. Lysozyme, which marks the presence of Paneth cells, was expressed at a low level on day 15 but was remarkably increased by day 30. The high expression levels were maintained over 50 days of culture. The expressions of Villin 1 (*VIL1*) (brush border of the enterocytes) and Chromogranin A (*CHGA*) (enteroendrocrine cells) peaked at day 30 and decreased later on as well. Finally, in both apical-in and apical-out organoids we identified the presence of mesenchyme. The distal hindgut mesoderm marker Homeobox A13 (*HOXA13*) peaked at day 15 and gradually decreased over later time-points. In contrast, the expression of the mesenchymal markers Forkhead Box F1 (*FOXF1*) and *VIM* remained unchanged between days 15 and 30, whereas on day 50 it was notably reduced. The expression patterns of the basal-out and apical-out organoids were very similar and no statistical significance was identified at any time-point (both in H9- and iPSC-derived organoids). These results indicate that organoids on day 15 already express intestine-specific markers but are fairly immature, whereas by day 30 the *in vitro* maturation culminates. This is in accordance with findings from Spence et al. (2011) and Janssen et al. (2020). After 50 days in culture, we observed a general decrease in the expression of most markers, although still in detectable levels, showing that organoids’ functionality in culture gradually deteriorates from day 30 days. Engraftment of organoids in mice would secure further maturation (Watson et al., 2014), overcoming the short-term culturing periods allowed by *in vitro* methods. However, it is unknown whether the apical-out organoids would be able to maintain their polarity.

## Discussion

In recent years, the advent of intestinal organoids has revolutionized the *in vitro* research of the intestinal epithelium. These organoids have been widely used in studies related to gut development, physiology and disease since they recapitulate the properties of the *in vivo* tissue with great fidelity. In the original method, the intestinal organoids demonstrate an organized structure, where the basal side is in contact with the ECM and facing outwards whereas the apical side is enclosed and facing the luminal compartment. Thus, access to the apical surface is restricted and microinjection techniques are required to deliver substances (Bartfeld et al., 2015; Hill et al., 2017; Williamson et al., 2018). Recent advances in the organoid field paved the way for easier access to the lumen by reversing epithelial polarity in adult stem cell-derived organoids (Co et al., 2019; Li et al., 2020; Co et al., 2021; Giobbe et al., 2021; Nash et al., 2021), but whether the same method could be applied in PSC-derived organoids was uncertain. Here, we developed and validated a reversed polarity organoid model using PSCs. In these organoids the apical side is found on the outer surface of the organoids, and, thus, is directly accessible for testing compounds, particles or microbes. Apical-out organoids demonstrate similar functionality to the basal-out organoids as suggested by the retention of self-renewal capacity throughout the whole culture and the expression of all major intestinal cell types. One advantage of the apical-out organoids is that they are grown in a suspension system. In this way, the handling of organoids is uncomplicated since there is no viscous hydrogel surrounding them. This suggests that organoids can be easily selected and (re-)transferred, e.g., into microwells for performing downstream experiments.

The intestinal epithelium acts as a highly selective barrier for the absorption, metabolism and release of nutrients and drugs. Currently, the investigation of these functions is mainly performed with cell monolayers (Transwell systems) and there are only few examples of organoid applications (Zietek et al., 2020; Youhanna and Lauschke, 2021). This is mainly due to the inaccessibility of the apical surface of the organoids. The formation of cell monolayers, either using cell lines (e.g., Caco-2) (Sun et al., 2008) or dissociated organoids (VanDussen et al., 2015; Kozuka et al., 2017; Wang et al., 2017; Altay et al., 2019) provides access to both the apical and basal sides. However, in this case a large number of cells is required and usually several days of maturation. Also, the 3D organization is disrupted, thus making the system less physiologically relevant. Another limitation is that usually these monolayers are formed in Transwell systems, which restricts *in situ* monitoring, e.g., live cell imaging, during culture. In contrast to this, our apical-out intestinal organoids, both retain their 3D architecture and allow for easy tracking and monitoring throughout the culture period. Hence, apical-out organoids may represent a novel and improved model for nutrient uptake and drug absorption studies.

Although intestinal organoids constitute one of the most physiologically representative *in vitro* models, the integral gut microbiome is missing (Min et al., 2020). In order to incorporate this in organoid models, researchers utilize either microinjections or monolayer cultures. Reversing the polarity of organoids offers an easy access to the apical surface, in which the microbiota is residing *in vivo*. Hence, with this system, host-microbiome interactions can be studied simply by adding microorganisms in the culture medium of organoids. The same method can be applied for the study of host-pathogen interactions where unknown mechanisms of cell invasion can be explored, thus leading to novel, efficient therapies. First successful experiments with apical-out organoids and microorganisms have already been performed by Co et al. (2019), Li et al. (2020), Nash et al. (2021), but so far solely adult stem cell-derived organoids could be used for this. In case of PSC-derived organoids, microbial colonization and pathogen infections can be studied at different stages of development, which is particularly important since the early stages of gut microbiota development remains poorly understood (Senn et al., 2020).

In summary, apical-out intestinal organoids can be successfully generated in microwell arrays, from PSC-derived 3D EBs following a step-wise differentiation method. These organoids reflect the structural and functional characteristics of their *in vivo* counterparts. The long-term reversed polarity grants easy access to the apical compartment thus qualifying these organoids for a wide range of applications.

## Data Availability

The original contributions presented in the study are included in the article/Supplementary Material, further inquiries can be directed to the corresponding author.

## References

[B1] Altay G. Larrañaga E. Tosi S. Barriga F. M. Batlle E. Fernández-Majada V. (2019). Self-organized Intestinal Epithelial Monolayers in Crypt and Villus-like Domains Show Effective Barrier Function. Sci. Rep. 9 (9), 1–14. 10.1038/s41598-019-46497-x 31300688 PMC6625996

[B2] Bartfeld S. Bayram T. Van De Wetering M. Huch M. Begthel H. Kujala P. (2015). *In Vitro* expansion of Human Gastric Epithelial Stem Cells and Their Responses to Bacterial Infection. Gastroenterology 148, 126–136. 10.1053/j.gastro.2014.09.042 25307862 PMC4274199

[B3] Caradec J. Sirab N. Keumeugni C. Moutereau S. Chimingqi M. Matar C. (2010). 'Desperate House Genes': the Dramatic Example of Hypoxia. Br. J. Cancer 102, 1037–1043. 10.1038/sj.bjc.6605573 20179706 PMC2844028

[B4] Clevers H. (2016). Modeling Development and Disease with Organoids. Cell 165, 1586–1597. 10.1016/J.CELL.2016.05.082 27315476

[B5] Co J. Y. Margalef-Català M. Li X. Mah A. T. Kuo C. J. Monack D. M. (2019). Controlling Epithelial Polarity: A Human Enteroid Model for Host-Pathogen Interactions. Cel Rep. 26, 2509–2520. 10.1016/j.celrep.2019.01.108 PMC6391775 30811997

[B6] Co J. Y. Margalef-Català M. Monack D. M. Amieva M. R. (2021). Controlling the Polarity of Human Gastrointestinal Organoids to Investigate Epithelial Biology and Infectious Diseases. Nat. Protoc. 16 (16), 5171–5192. 10.1038/s41596-021-00607-0 34663962 PMC8841224

[B7] Dessimoz J. Opoka R. Kordich J. J. Grapin-Botton A. Wells J. M. (2006). FGF Signaling Is Necessary for Establishing Gut Tube Domains Alongthe Anterior-Posterior axis *In Vivo* . Mech. Develop. 123, 42–55. 10.1016/J.MOD.2005.10.001 16326079

[B8] Fatehullah A. Tan S. H. Barker N. (2016). Organoids as an *In Vitro* Model of Human Development and Disease. Nat. Cel Biol. 18, 246–254. 10.1038/ncb3312 26911908

[B9] Giobbe G. G. Bonfante F. Jones B. C. Gagliano O. Luni C. Zambaiti E. (2021). SARS-CoV-2 Infection and Replication in Human Gastric Organoids. Nat. Commun. 12, 1–14. 10.1038/s41467-021-26762-2 34785679 PMC8595698

[B10] Giselbrecht S. Gietzelt T. Gottwald E. Trautmann C. Truckenmüller R. Weibezahn K. F. (2006). 3D Tissue Culture Substrates Produced by Microthermoforming of Pre-processed Polymer Films. Biomed. Microdevices 8, 191–199. 10.1007/s10544-006-8174-8 16718404

[B11] Hill D. R. Huang S. Tsai Y.-H. Spence J. R. Young V. B. (2017). Real-time Measurement of Epithelial Barrier Permeability in Human Intestinal Organoids. JoVE 130, e56960. 10.3791/56960 PMC5755602 29286482

[B12] Janssen A. W. F. Duivenvoorde L. P. M. Rijkers D. Nijssen R. Peijnenburg A. A. C. M. van der Zande M. (2020). Cytochrome P450 Expression, Induction and Activity in Human Induced Pluripotent Stem Cell-Derived Intestinal Organoids and Comparison with Primary Human Intestinal Epithelial Cells and Caco-2 Cells. Arch. Toxicol. 95, 907–922. 10.1007/S00204-020-02953-6 33263786 PMC7904554

[B13] Kakni P. Hueber R. Knoops K. López‐Iglesias C. Truckenmüller R. Habibovic P. (2020). Intestinal Organoid Culture in Polymer Film‐Based Microwell Arrays. Adv. Biosys. 4, 2000126. 10.1002/adbi.202000126 32734713

[B14] Klunder L. J. Faber K. N. Dijkstra G. van IJzendoorn S. C. D. (2017). Mechanisms of Cell Polarity-Controlled Epithelial Homeostasis and Immunity in the Intestine. Cold Spring Harb. Perspect. Biol. 9, a027888. 10.1101/cshperspect.a027888 28213466 PMC5495056

[B15] Kozuka K. He Y. Koo-McCoy S. Kumaraswamy P. Nie B. Shaw K. (2017). Development and Characterization of a Human and Mouse Intestinal Epithelial Cell Monolayer Platform. Stem Cel Rep. 9, 1976–1990. 10.1016/J.STEMCR.2017.10.013 PMC5785676 29153987

[B16] Li Y. Yang N. Chen J. Huang X. Zhang N. Yang S. (2020). Next-Generation Porcine Intestinal Organoids: an Apical-Out Organoid Model for Swine Enteric Virus Infection and Immune Response Investigations. J. Virol. 94, e01006. 10.1128/JVI.01006-20 32796075 PMC7565635

[B17] McCracken K. W. Wells J. M. (2017). Mechanisms of Embryonic Stomach Development. Semin. Cel Develop. Biol. 66, 36–42. 10.1016/j.semcdb.2017.02.004 PMC5474362 28238948

[B18] McCracken K. W. Howell J. C. Wells J. M. Spence J. R. (2011). Generating Human Intestinal Tissue from Pluripotent Stem Cells *In Vitro* . Nat. Protoc. 6, 1920–1928. 10.1038/nprot.2011.410 22082986 PMC3896236

[B19] Min S. Kim S. Cho S.-W. (2020). Gastrointestinal Tract Modeling Using Organoids Engineered with Cellular and Microbiota Niches. Exp. Mol. Med. 52, 227–237. 10.1038/s12276-020-0386-0 32103122 PMC7062772

[B20] Nash T. J. Morris K. M. Mabbott N. A. Vervelde L. (2021). Inside-out Chicken Enteroids with Leukocyte Component as a Model to Study Host-Pathogen Interactions. Commun. Biol. 4, 1–15. 10.1038/s42003-021-01901-z 33742093 PMC7979936

[B21] Sato T. Clevers H. (2013). Growing Self-Organizing Mini-Guts from a Single Intestinal Stem Cell: Mechanism and Applications. Science 340, 1190–1194. 10.1126/science.1234852 23744940

[B22] Sato T. Vries R. G. Snippert H. J. Van De Wetering M. Barker N. Stange D. E. (2009). Single Lgr5 Stem Cells Build Crypt-Villus Structures *In Vitro* without a Mesenchymal Niche. Nature 459, 262–265. 10.1038/nature07935 19329995

[B23] Senn V. Bassler D. Choudhury R. Scholkmann F. Righini-Grunder F. Vuille-dit-Bile R. N. (2020). Microbial Colonization from the Fetus to Early Childhood-A Comprehensive Review. Front. Cel. Infect. Microbiol. 10, 573735. 10.3389/FCIMB.2020.573735/BIBTEX PMC7661755 33194813

[B24] Spence J. R. Mayhew C. N. Rankin S. A. Kuhar M. F. Vallance J. E. Tolle K. (2011). Directed Differentiation of Human Pluripotent Stem Cells into Intestinal Tissue *In Vitro* . Nature 470, 105–109. 10.1038/nature09691 21151107 PMC3033971

[B25] Stroulios G. Stahl M. Elstone F. Chang W. Louis S. Eaves A. (2021). Culture Methods to Study Apical-specific Interactions Using Intestinal Organoid Models. JoVE 2021, e62330. 10.3791/62330 33843928

[B26] Sun H. Chow E. C. Y. Liu S. Du Y. Pang K. S. (2008). The Caco-2 Cell Monolayer: Usefulness and Limitations. Expert Opin. Drug Metab. Toxicol. 4, 395–411. 10.1517/17425255.4.4.395 18433344

[B27] VanDussen K. L. Marinshaw J. M. Shaikh N. Miyoshi H. Moon C. Tarr P. I. (2015). Development of an Enhanced Human Gastrointestinal Epithelial Culture System to Facilitate Patient-Based Assays. Gut 64, 911–920. 10.1136/GUTJNL-2013-306651 25007816 PMC4305344

[B28] Wang Y. DiSalvo M. Gunasekara D. B. Dutton J. Proctor A. Lebhar M. S. (2017). Self-renewing Monolayer of Primary Colonic or Rectal Epithelial Cells. Cell Mol. Gastroenterol. Hepatol. 4, 165–182. 10.1016/J.JCMGH.2017.02.011 29204504 PMC5710741

[B29] Watson C. L. Mahe M. M. Múnera J. Howell J. C. Sundaram N. Poling H. M. (2014). An *In Vivo* Model of Human Small Intestine Using Pluripotent Stem Cells. Nat. Med. 20, 1310–1314. 10.1038/nm.3737 25326803 PMC4408376

[B30] Williamson I. A. Arnold J. W. Samsa L. A. Gaynor L. DiSalvo M. Cocchiaro J. L. (2018). A High-Throughput Organoid Microinjection Platform to Study Gastrointestinal Microbiota and Luminal Physiology. Cell Mol. Gastroenterol. Hepatol. 6, 301–319. 10.1016/j.jcmgh.2018.05.004 30123820 PMC6092482

[B31] Youhanna S. Lauschke V. M. (2021). The Past, Present and Future of Intestinal *In Vitro* Cell Systems for Drug Absorption Studies. J. Pharm. Sci. 110, 50–65. 10.1016/J.XPHS.2020.07.001 32628951

[B32] Zietek T. Giesbertz P. Ewers M. Reichart F. Weinmüller M. Urbauer E. (2020). Organoids to Study Intestinal Nutrient Transport, Drug Uptake and Metabolism - Update to the Human Model and Expansion of Applications. Front. Bioeng. Biotechnol. 8, 1065. 10.3389/FBIOE.2020.577656 PMC7516017 33015026

[B33] Zorn A. M. Wells J. M. (2009). Vertebrate Endoderm Development and Organ Formation. Annu. Rev. Cel Dev. Biol. 25, 221–251. 10.1146/annurev.cellbio.042308.113344 PMC2861293 19575677

