# Supplementary material for: Reversing Epithelial Polarity in Pluripotent Stem Cell-Derived Intestinal Organoids
Source: Front Bioeng Biotechnol. 2022 Apr 25;10:879024. doi: 10.3389/fbioe.2022.879024 (PMC9081652; doi:10.3389/fbioe.2022.879024)
Supplement: Supplementary file 1 [file DataSheet1.pdf]

Supplementary Material

Reversing epithelial polarity in pluripotent stem cell-derived intestinal organoids

Panagiota Kakni[1], Carmen López-Iglesias[2], Roman Truckenmüller[1], Pamela Habibovic[1], and Stefan Giselbrecht[1]*

1 Supplementary Tables

1.1 Supplementary Table 1. List of antibodies

Antibodies	Supplier	Host	Dilution
Oct-3/4	Santa Cruz	Mouse	1:500
Nanog	Abcam	Rabbit	1:500
E-Cadherin	Beckton Dickinson	Mouse	1:500
FOXA2	Novus Bio	Mouse	1:1000
SOX17	R&D	Goat	1:500
Ki67	Abcam	Rabbit	1:500
CDX2	Biogenex	Mouse	1:500
MUC2	Abcam	Rabbit	1:250
Synaptophysin 1	Synaptin systems	Guinea pig	1:200
Villin	Santa cruz	Mouse	1:250
Phalloidin 568	Invitrogen		1:500
Alexa Fluor 488	Invitrogen		1:500
Alexa Fluor 647	Invitrogen		1:500
Alexa Fluor 568	Invitrogen		1:500

1.2 Supplementary Table 2. Primer sequences

Gene	5'–Forward– 3'	5'–Reverse– 3'
ALB	TGCAACTCTTCGTGAAACCTATG	ACATCAACCTCTGGTCTCACC
ASCL2	GCGTGAAGCTGGTGAACTTG	GGATGTACTCCACGGCTGAG
CDX2	GACGTGAGCATGTACCCTAGC	GCGTAGCCATTCCAGTCCT
CHGA	TAAAGGGGATACCGAGGTGATG	TCGGAGTGTCTCAAAACATTCC
FOXA2	CGACTGGAGCAGCTACTATGC	TACGTGTTCATGCCGTTCAT
FOXF1	GCGGCTTCCGAAGGAAATG	CAAGTGGCCGTTCATCATGC
GAPDH	GGAGCGAGATCCCTCCAAAAT	GGCTGTTGTCATACTTCTCATGG
HOXA13	CTGCCCTATGGCTACTTCGG	CCGGCGGTATCCATGTACT
HPRT	GGACTCCAGATGTTTCCAAACTC	TTGTTGTAGGATATGCCCTTGAC
KLF5	CCTGGTCCAGACAAGATGTGA	GAACTGGTCTACGACTGAGGC
LGR5	CTCCCAGGTCTGGTGTGTTG	GAGGTCTAGGTAGGAGGTGAAG
LYZ	TCAATAGCCGCTACTGGTGTA	ATCACGGACAACCCTCTTTGC
SOX17	GTGGACCGCACGGAATTTG	GGAGATTCACACCGGAGTCA
SOX9	AGCGAACGCACATCAAGAC	CTGTAGGCGATCTGTTGGGG
TBXT	TATGAGCCTCGAATCCACATAGT	CCTCGTTCTGATAAGCAGTCAC
VIL1	CTGAGCGCCCAAGTCAAAG	AGCAGTCACCATCGAAGAAGC
VIM	GACGCCATCAACACCGAGTT	CTTTGTCGTTGGTTAGCTGGT

2 Supplementary Figures

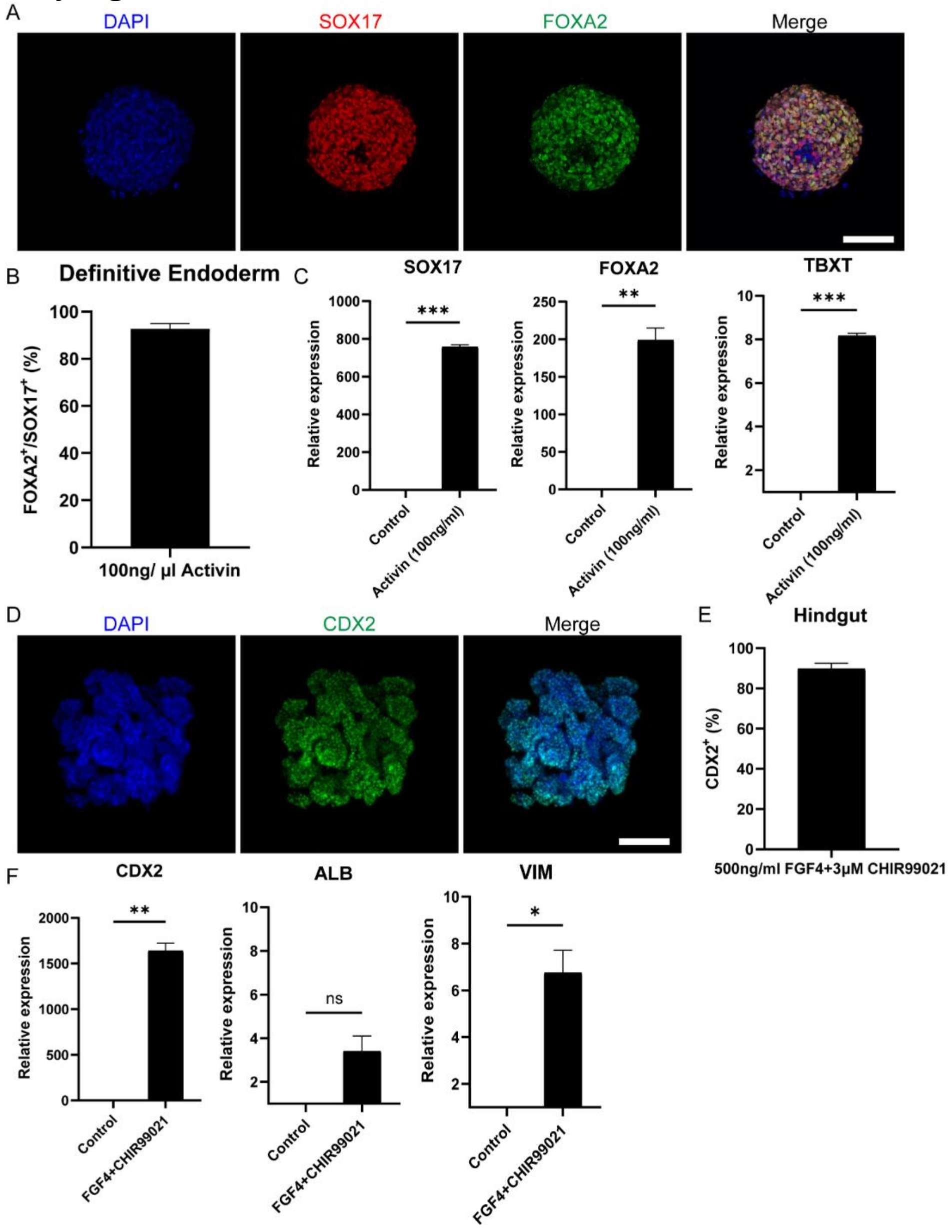

Supplementary Figure 1. Differentiation towards definitive endoderm followed by hindgut specification. **(A)** iPSC72_3-derived EBs were treated with 100ng/ml Activin and the resulting spheroids were stained with the DE markers: SOX17 (red) and FOXA2 (green) and counterstained with DAPI (blue). Scale bar: 100µm. **(B)** Quantification of the fluorescent images showed that about 90% of the cells in Activin-treated EBs are co-expressing SOX17 and FOXA2. **(C)** qRT-PCR showed significantly increased expression of the DE genes SOX17 and FOXA2 and the mesoderm marker TBXT but in lower amounts. **(D)** DE spheroids were further treated with FGF4 and CHIR99021 to induce hindgut specification. After 4 days of treatment, the spheroids were stained for the hindgut marker CDX2. **(E)** Quantification of the fluorescent images showed that about 91% of the cells were CDX2^{+}. **(F)** qRT-PCR confirmed the robust expression of CDX2, whereas there was no significant expression of the foregut marker ALB. Low levels of the mesenchymal marker VIM were also detected. Error bars indicate mean ± S.E.M. (n = 3).

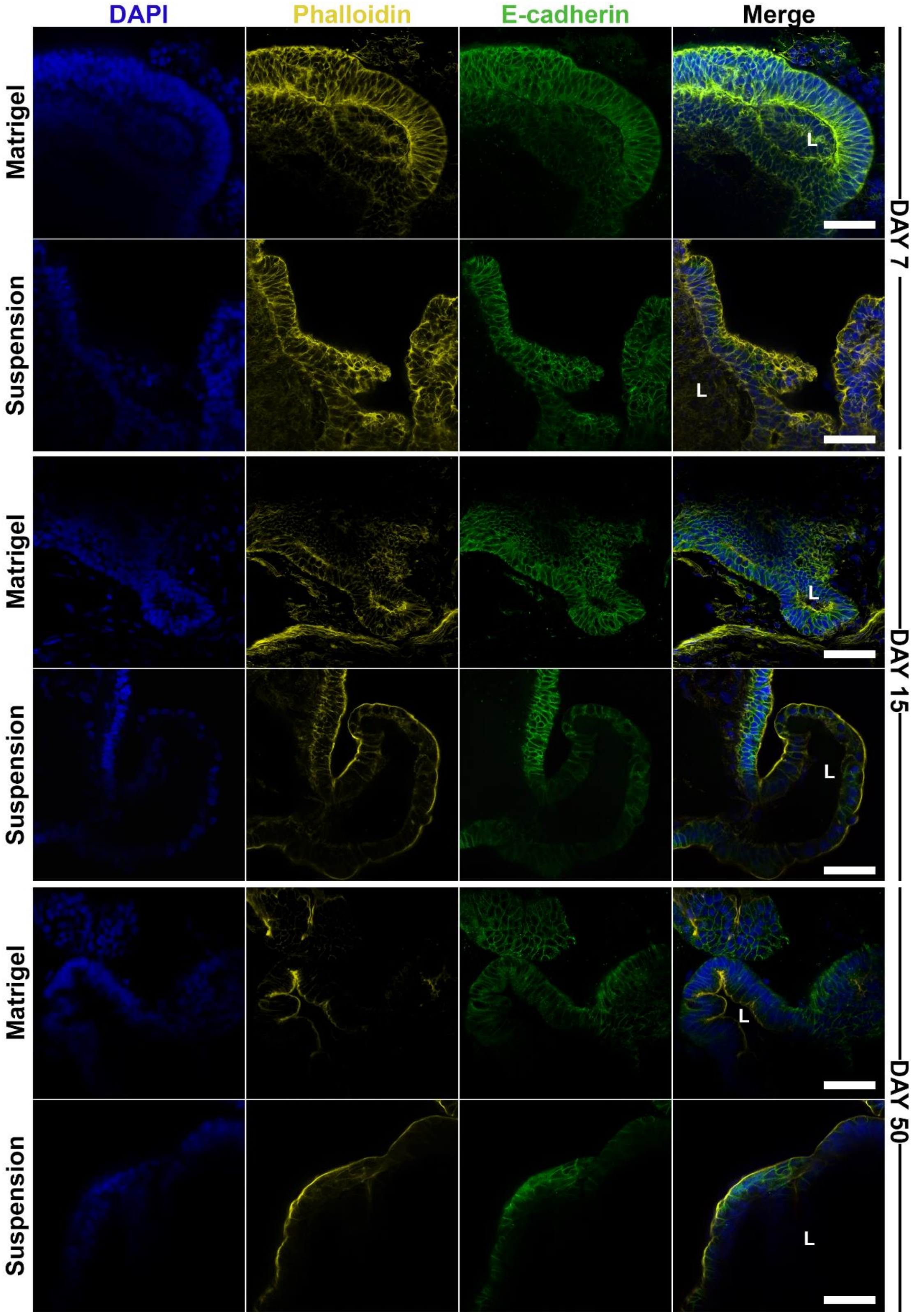

Supplementary Figure 2. Apico-basolateral polar organization in different time-points. **(A)** H9-derived human intestinal organoids present reversed polarity (apical side out) already after 7 days in culture as shown by immunofluorescence staining with E-cadherin (green) and Phalloidin (yellow).

Scale bar: 100μm. **(B-C)** Confocal imaging showed that these organoids maintain this organization after 15 (B) and even 50 days (C) in culture. Scale bar: 100μm.

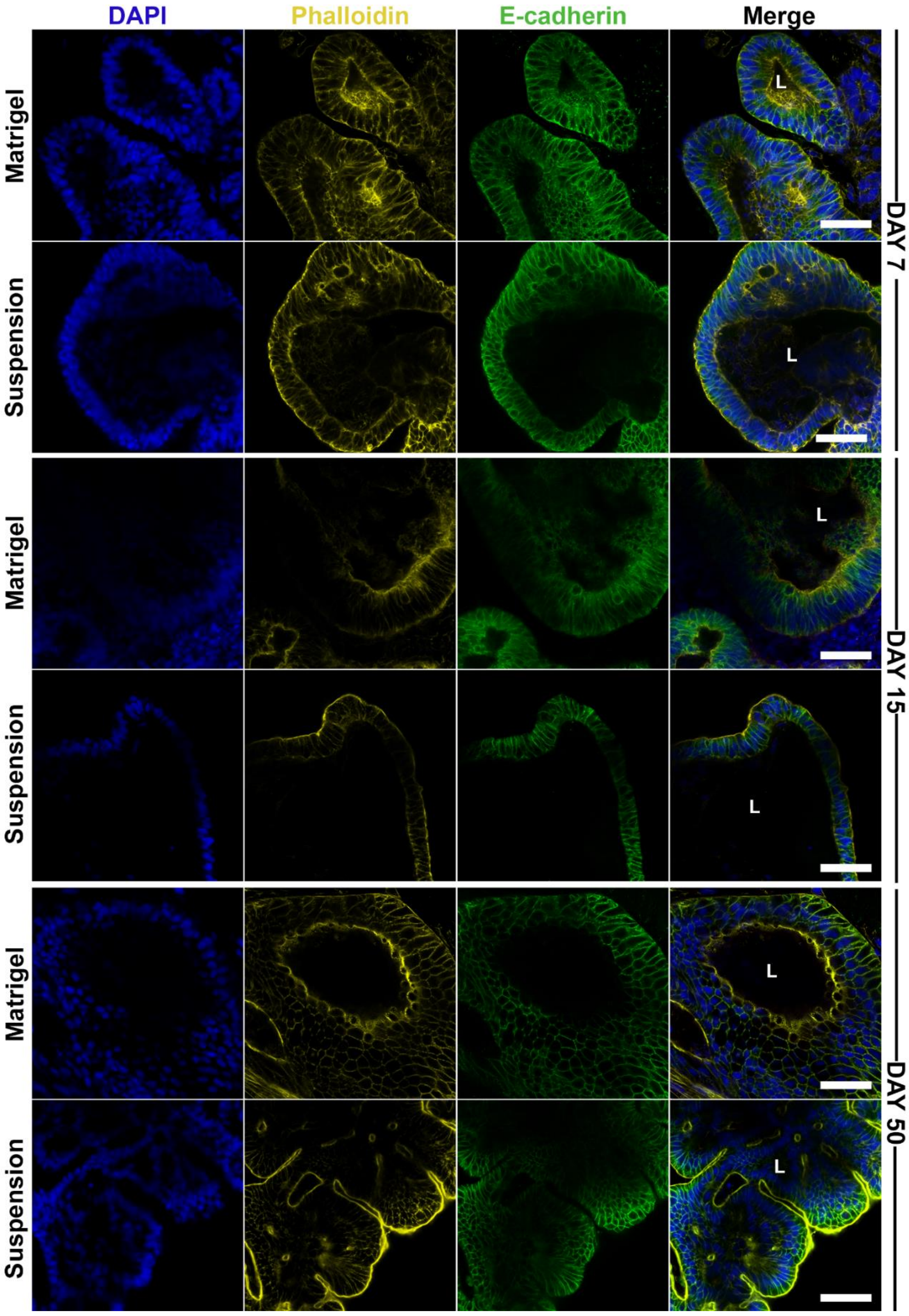

Supplementary Figure 3. Apico-basolateral polar organization in different time-points. **(A)** iPSC72_3-derived human intestinal organoids present reversed polarity (apical side out) already after 7 days in culture as shown by immunofluorescence staining with E-cadherin (green) and Phalloidin (yellow). Scale bar: 100µm. **(B-C)** Confocal imaging showed that these organoids maintain this organization after 15 (B) and even 50 days (C) in culture. Scale bar: 100µm.

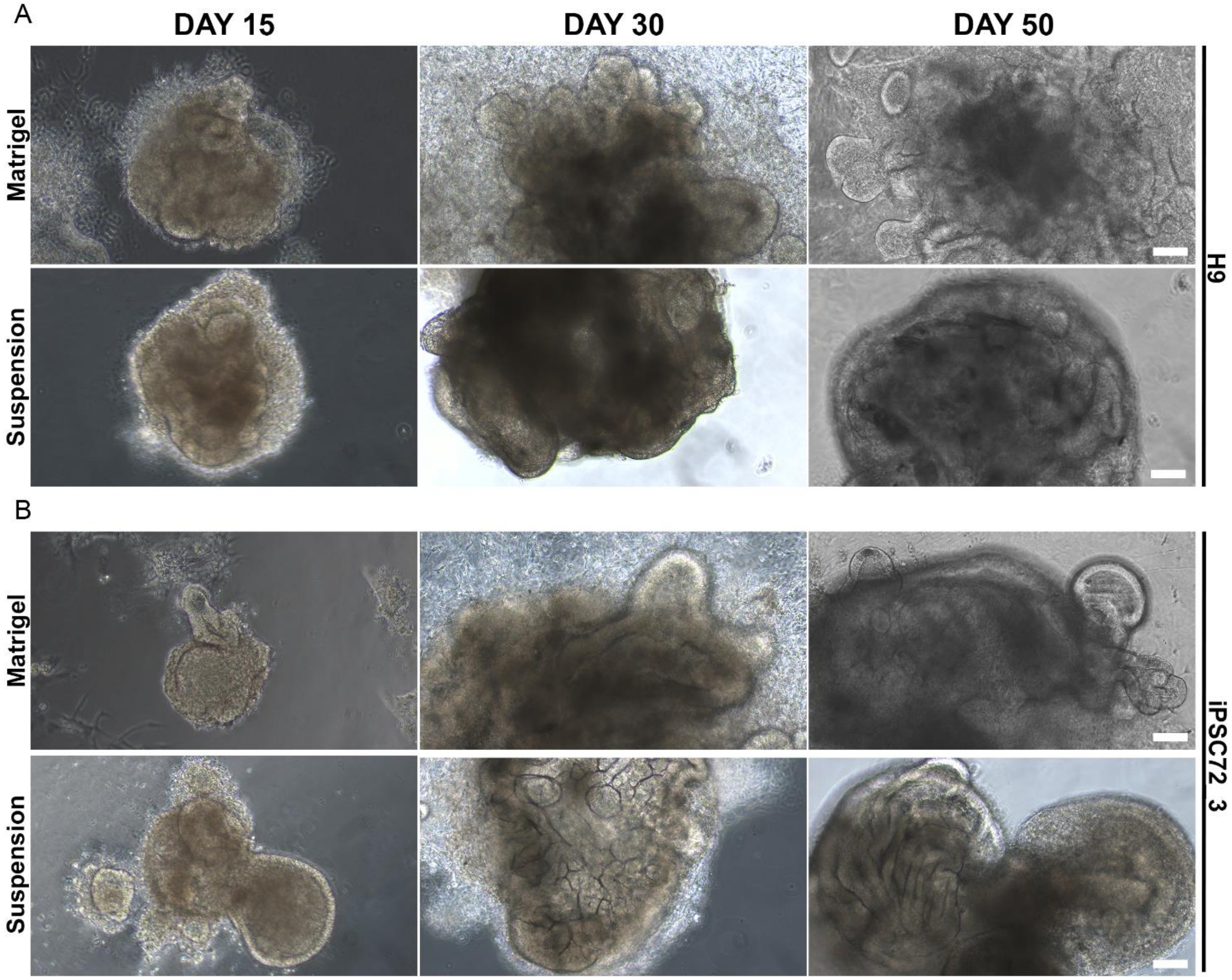

Supplementary Figure 4. Organoid morphology during culture. **(A-B)** Bright-field images demonstrating the growth and morphology of intestinal organoids after 15, 30 and 50 days in culture. The top panel indicates the H9-derived organoids (A) and the bottom one the iPSC72_3-derived organoids (B). Scale bar: 200µm.

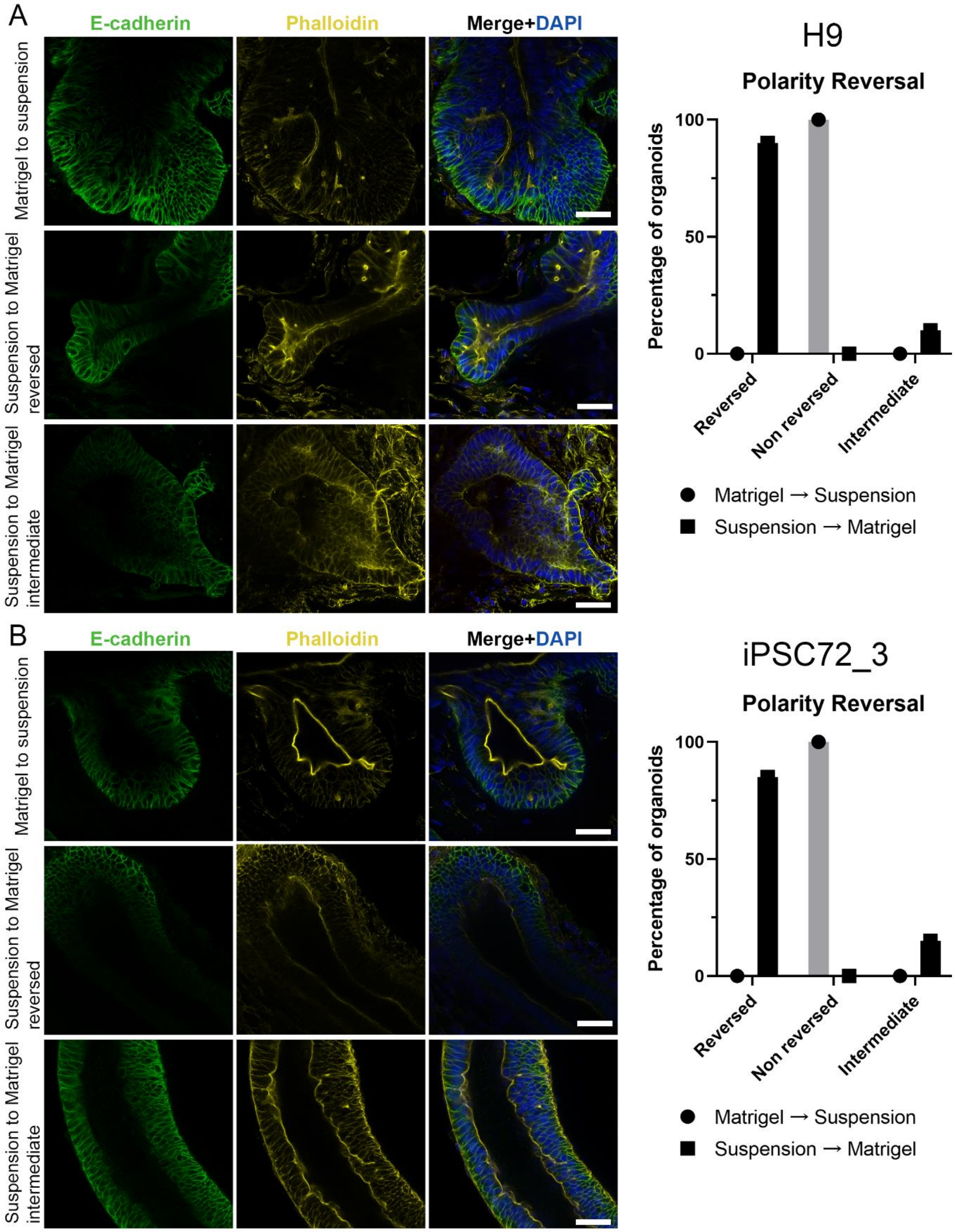

Supplementary Figure 5. Polarity reversal plasticity. **(A)** H9-derived organoids: Immunofluorescence stainings with E-cadherin (green) and Phalloidin (yellow) demonstrate that Matrigel embedded organoids that are placed in suspension culture for 7 days do not reverse polarity (top). When suspension organoids are embedded in Matrigel, the polarity is reversing in 90% of the organoids and

the apical side is facing the organoid lumen (middle). The rest 10% is demonstrating an intermediate organization (bottom). Scale bars: 100μm. (n=4). **(B)** iPSC72_3-derived organoids: Immunofluorescence stainings with E-cadherin (green) and Phalloidin (yellow) demonstrate that similar to H9-derived organoids Matrigel embedded organoids that are placed in suspension culture for 7 days do not reverse polarity (top). When suspension organoids are embedded in Matrigel, the polarity is reversing in 85% of the organoids and the apical side is facing the organoid lumen (middle). The rest 15% is demonstrating an intermediate organization (bottom). Scale bars: 100μm. (n=4).

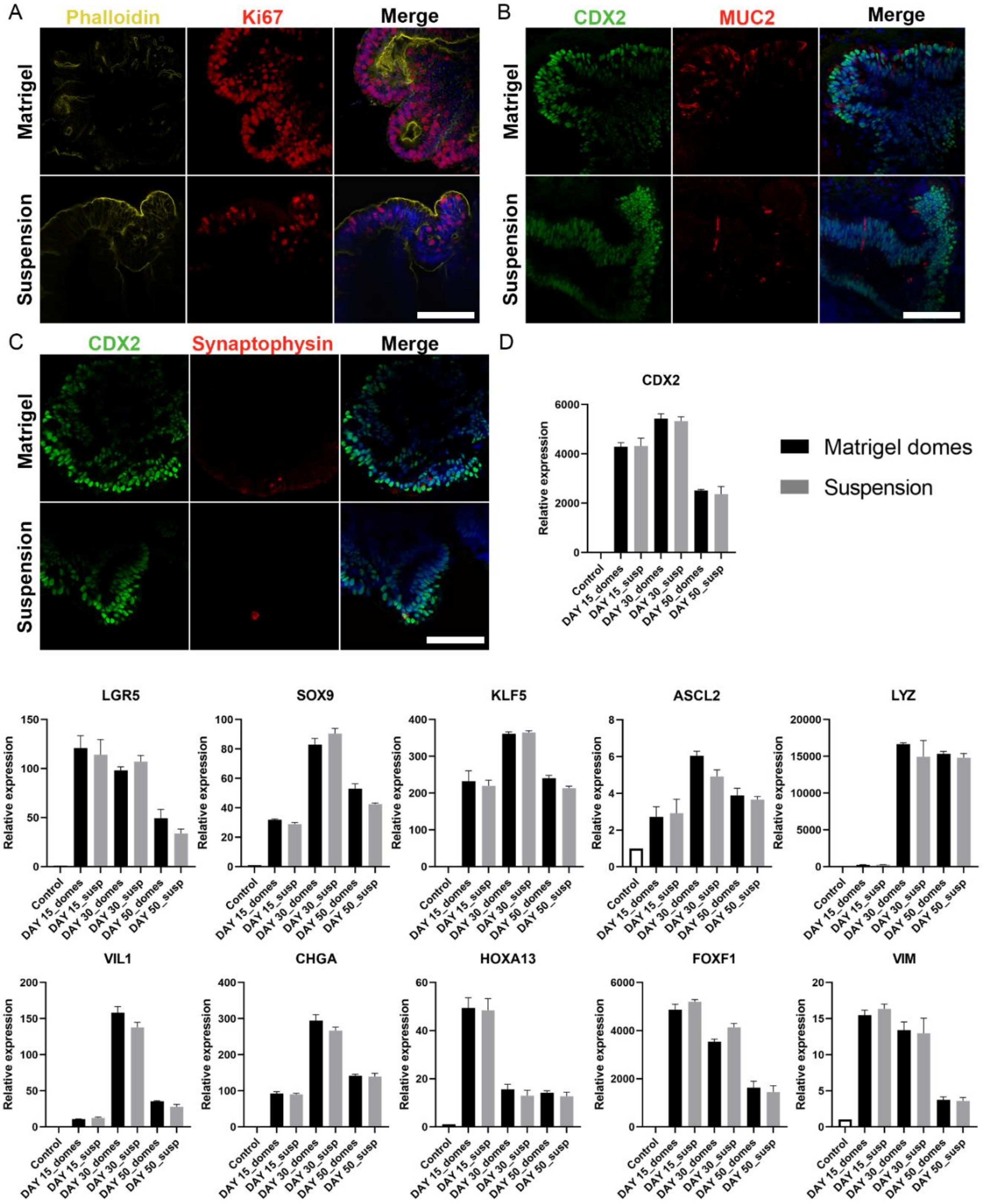

Supplementary Figure 6. Characterization of iPSC72_3-derived human intestinal organoids after 30 days in culture. **(A-C)** Immunofluorescence stainings of intestinal markers (Ki67: proliferative cells; CDX2: hindgut; MUC2: goblet cells; Synaptophysin: enteroendrocrine cells) show similar expression patterns in both embedded and suspension organoids. Scale bars: 100µm. **(D)** qRT-PCR analysis demonstrates the expression levels of proliferation genes (*LGR5*, *SOX9*, *KLF5*, *ASCL2*), intestinal differentiation genes (*CDX2*, *LYZ*, *VIL1*, *CHGA*, *HOXA13*) and mesenchymal genes (*FOXF1*, *VIM*) after 15, 30 and 50 days in culture. Untreated iPSC72_3 cells were used as controls. Statistical analysis showed no significant difference between the organoids grown embedded in Matrigel and the organoids grown in suspension at any of the time-points. Error bars indicate mean ± S.E.M. (n = 3).
